# Validation and reliability of a guideline appraisal mini-checklist for daily practice use

**DOI:** 10.1186/s12874-016-0139-x

**Published:** 2016-04-02

**Authors:** Andrea Siebenhofer, Thomas Semlitsch, Thomas Herborn, Ulrich Siering, Ina Kopp, Johannes Hartig

**Affiliations:** Institute of General Practice and Evidence-Based Health Services Research, Medical University of Graz, Auenbruggerplatz 2/9, 8036 Graz, Austria; Institute of General Practice, Goethe University Frankfurt, Frankfurt, Germany; Institute for Quality and Efficiency in Health Care (IQWiG), Cologne, Germany; Association of Scientific Medical Societies’ Institute of Medical Knowledge Management (AWMF-IMWi), Marburg, Germany; Department of Educational Quality and Evaluation, German Institute for International Educational Research (DIPF), Frankfurt, Germany

**Keywords:** Guideline assessment, Validation, Reliability, AGREE II instrument, Mini-checklist, General practitioners

## Abstract

**Background:**

The use of comprehensive instruments for guideline appraisal is time-consuming and requires highly qualified personnel. Since practicing physicians are generally busy, the rapid-assessment Mini-Checklist (MiChe) tool was developed to help them evaluate the quality and utility of guidelines quickly. The aim of this study was to validate the MiChe in comparison to the AGREE II instrument and to determine its reliability as a tool for guideline appraisal.

**Methods:**

Ten guidelines that are relevant to general practice and had been evaluated by 2 independent reviewers using AGREE II were assessed by 12 GPs using the MiChe. The strength of the correlation between average MiChe ratings and AGREE II total scores was estimated using Pearson’s correlation coefficient. Inter-rater reliability for MiChe overall quality ratings and endorsements was determined using intra-class correlations (ICC) and Kendall’s W for ordinal recommendations. To determine the GPs’ satisfaction with the MiChe, mean scores for the ratings on five questions were computed using a six-point Likert scale.

**Results:**

The study showed a high level of agreement between MiChe and AGREE II in the quality rating of guidelines (Pearson’s *r* = 0.872; *P* < 0.001). Inter-rater-reliability for overall MiChe ratings (ICC = 0.755; *P* < 0.001) and endorsements (Kendall’s W = 0.73; *P* < 0.001) were high. The mean time required for guideline assessment was less than 15 min und user satisfaction was generally high.

**Conclusions:**

The MiChe performed well in comparison to AGREE II and is suitable for the rapid evaluation of guideline quality and utility in practice.

**Trial registration:**

German Clinical Trials Register: DRKS00007480

## Background

Clinical practice guidelines are defined by the Institute of Medicine as “statements that include recommendations intended to optimize patient care that are informed by a systematic review of evidence and an assessment of the benefits and harms of alternative care options” [1]. There is evidence to suggest that rigorously developed guidelines have the power to translate the complexity of scientific research findings and other evidence into recommendations for healthcare action [2–8]. To increase guideline quality, several institutions [1, 9–23] have prepared manuals, that attempt to define standards for guideline developers. At the same time, tools have been developed to help potential guideline users to assess guideline quality. The most commonly used international guideline appraisal tool is the AGREE II Instrument [24], but its use is time consuming and demands considerable skill on the part of the guideline appraiser.

Graham 2000 identified and compared guideline appraisal tools in a systematic review [25], which was later updated by Vlayen in 2005 [26] and Siering in 2013 [27]. Siering identified 40 different appraisal tools that vary considerably in terms of the number of quality dimensions taken into account. In the opinion of the authors, appraisal tools containing many quality dimensions may not represent the best choice in all cases. Depending on the problem being addressed, a tool containing a few well thought out questions may well suffice.

To be effective, guidelines must be applied by clinicians. An appraisal tool that is quick and easy to use and assesses the most relevant quality dimensions of a guideline would generally encourage their wider use. We therefore developed and published a mini-checklist (MiChe) for the rapid appraisal of the usefulness and quality of a guideline for clinical practitioners. Detailed information on the development process is provided elsewhere [28]. However, the development was based on a systematic search in guideline directories and bibliographic databases for guideline appraisal instruments. The assessment criteria used in the retrieved instruments were identified, and their importance to the development of an effective rapid assessment tool was judged by German guideline experts. The key criteria for MiChe were then selected on the basis of the most commonly found criteria in the retrieved instruments and the ratings from the expert survey.

Our primary objective was to validate the MiChe vs. the AGREE II instrument and determine its reliability for daily users in terms of ability to rapidly assess the strengths and weaknesses of a guideline and dependability of content.

## Methods

Twelve general practitioners (GPs) were asked to use the MiChe to assess 10 eligible guidelines that had already been evaluated by 2 independent reviewers using the AGREE II instrument.

### Aims

Primary outcomes

- Validate the overall quality rating of AGREE II as the gold standard vs. the overall quality rating of the MiChe.
- Estimate the inter-rater reliability of the overall quality rating assigned by different guideline assessors using the MiChe.

Secondary outcomes relating to the MiChe alone:

- Demonstrate the inter-rater reliability of endorsement: willing to recommend this guideline for use in practice (“yes”; “yes, with certain reservations” or “no”).
- Demonstrate user satisfaction to indicate whether the MiChe would help raters decide whether to use a specific guideline or not.
- Feedback to improve the MiChe.
- Time required for an assessment using MiChe.

Tertiary outcomes:

Evaluate the correlation between overall quality rating and endorsement of the MiChe vs. quality ratings of individual items (domain 1 – 6) of AGREE II.

### Participants

During a quality circle that took place in November 2014, a convenience sample of GPs working as resident doctors was recruited from the more than 100 accredited general practices that make up GP Research Network Frankfurt (ForN) [29]. GPs with experience of guideline development or appraisal, i.e. members of guideline commissions or GPs in postgraduate training were excluded. All participants received 1.5-h of training on the basics of guideline development and appraisal at the Institute of General Practice in Frankfurt. In addition, a sample guideline was provided, along with instructions to read and appraise the guideline using the MiChe. Participants later received a folder with a printed version of 10 guidelines and were asked to use the MiChe to appraise them. Results were returned by mail.

In the Federal State of Hesse, Germany, the code of medical ethics allows formal ethical approval to be waived upon request if the biomedical research to be conducted on patients or healthy volunteers involves no risky procedures and is not invasive. We contacted the local ethics committee of Frankfurt University Hospital, who informed us that ethical approval could be waived. As we were not expecting ethical approval to be required, participating GPs were only required to provide their verbal consent before starting to review the guidelines.

### Guideline selection and guideline assessment tools

The selection process was initiated by choosing guidelines already known to the study team and by studying a list of 20 guidelines, sorted according to their characteristics. Of these, 10 guidelines were selected that covered subjects that are relevant to general practice, had varying AGREE II quality levels, varied in length and were written in either German or English. Two independent reviewers with professional expertise in guideline appraisal from the Institute for Quality and Efficiency in Health Care (IQWiG) first assessed the guidelines using the AGREE II instrument. These assessments served as the gold standard for the validation [24].

**Fig. 1:**
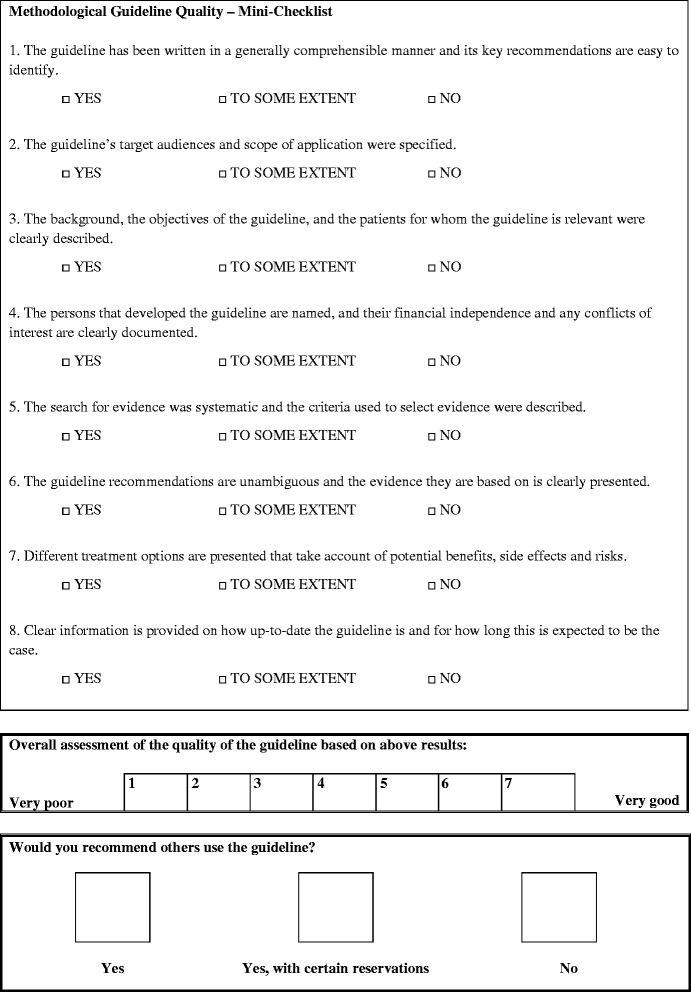
Mini-Checklist (MiChe)

The MiChe [28, 30] contains 8 key-criteria that focus on important methodological features (quality of guideline creation, quality of reporting, quality of presentation, quality of evidence synthesis), as well as a 3-level assessment scale (see Fig. 1).

### Data management

The GPs had to complete 10 MiChes for the 10 different guidelines and a short questionnaire on their personal characteristics and previous experience of guidelines. To indicate whether the MiChe would help raters to decide whether or not to use a guideline, 5 questions addressed user satisfaction (satisfaction, frequency of future use, makes it easier to deal with guidelines, influence of guideline recommendations on future daily practice use, comprehensibility) using a six-point Likert scale from 1 – 6, with 1 indicating a strong positive response. The average time required for assessment was measured separately for each guideline and GP. Suggestions for improvement and notes were documented in a free text field.

Ethics approval was not required, since no patients were involved. The protocol for this validity and reliability study was registered in the German Clinical Trials Register: DRKS00007480.

### Data analyses

#### Validity

The strength of the correlation between the average MiChe ratings of the guidelines and the AGREE II total score were estimated using Pearson’s correlation coefficient. A correlation of more than 0.70 is considered desirable. Additionally, correlations between the average recommendation on the MiChe and the separate AGREE II domains were calculated using Spearman’s rank order correlation.

#### Inter-rater reliability

Inter-rater agreement for the various MiChe ratings of the GPs was determined using intra-class correlations (ICC) and Kendall’s W for ordinal recommendations for endorsement (“yes”, “yes, with certain reservations”, “no”). For both coefficients, we consider values over 0.75 as good, values between 0.40 and 0.75 as moderate, and values below 0.40 as poor [31].

#### Evaluation of the mini checklist

To determine the GPs’ satisfaction with the MiChe, mean scores for the ratings on the five questions quoted in the data management section were computed.

#### Determination of required sample size

The required sample size to estimate inter-rater agreement can be determined by defining a specific null hypothesis and a specific alternative hypothesis, and selecting a desired type I and type II error rate (α and β level) [32]. We chose to set ICC = 0.50 as the lowest acceptable agreement for the null hypothesis and an expected value of ICC = 0.75 for the alternative hypothesis. For α = 0.05 and β = 0.20, 10 GPs would have to evaluate 14 guidelines. The 10 guidelines evaluated in our study would still yield a statistical power between 60 % and 70 %, and, as the guidelines are not sampled randomly but selected to elicit high variation in the AGREE II and MiChe ratings, it would probably be higher. This, in turn, makes it more likely to statistically confirm high inter-rater agreement.

## Results

### Characteristics of the GPs and the tested guidelines

Twelve GPs (6 female) participated in our study. Their mean age was 53 (SD 7) years and mean professional experience as a GP 19 (SD 7) years; 6 worked in a joint practice and 7 in a rural area with less than 60,000 inhabitants. None of the participants had used a guideline assessment tool before, but all 12 GPS had previously used guidelines as a source of information (Table 1).

**Table 1:** Characteristics of participating general practitioners

Characteristics (*N* = 12)	
Age [mean (SD)]	53 (7.0)
Gender	
Male [%]	50
Female [%]	50
Geographical delimitation	
Urban [%]	42
Rural [%]	58
Medical specialization	
General practitioner (GP) [%]	67
Specialist in Internal Medicine practising as GP [%]	33
Professional experience	
Years in practice [mean (SD)]	25 (7.0)
Years as GP [mean (SD)]	19 (7.3)
Guideline experience	
Used guidelines as source of information [%]	100
Former usage of e.g. AGREE or German equivalent instrument [%]	0^a^

^a^ one participant declined to respond

The included guidelines were published between 2006 and 2013, and covered different areas of relevance to general practice. Six guidelines were in German and 4 in English. They differed in length from 4 to 278 pages. The overall quality of the guidelines as assessed by AGREE II varied between 2 and 6 points on the 7-point scale. Four of them received a recommendation of “yes”, 4 of “yes, with certain reservations” and 2 were given a “no” recommendation. The average MiChe overall quality score across the 12 GPs ranged from 2.4 (SD 1.0) to 6.7 (SD 0.7) for the 10 guidelines. Based on the MiChe assessment, 6 guidelines received a majority recommendation of “yes”, 1 of “yes, with certain reservations” and 3 were given a “no” recommendation by the majority of the GPs. The total AGREE II score was lower than the total MiChe score for 7 of the 10 guidelines [33–39] and higher for the remaining 3 [40–42]. The DEGAM guideline on heart failure [34] was ranked best overall by both instruments and 2 guidelines [39, 42] were poorly ranked by both assessment tools (Table 2).

**Table 2:** Mean overall rating scores for AGREE II and the MiChe

Guideline			AGREE II		MiChe		
Title/Leading Organization (Abbreviation)	Year of publication/pages/region/language	Topic	Overall quality	Recommendation for use	Overall quality [mean (SD)]	Recommendation for use [%]	Mean duration of appraisal [minutes]
DEGAM Leitlinie Nr. 9, Herzinsuffizienz [Guideline no. 9 cardiac failure]/Deutsche Gesellschaft für Allgemeinmedizin und Familienmedizin (DEGAM 2006) [34]	2006/278/GER/German	Cardiac failure	6	Yes	6.7 (0.65)	Yes: 92	12.1
						Yes, with certain reservations: 8	
						No: 0	
Guideline for the Management of Acute Bronchitis/Alberta	2008/6/CDN/English	Bronchitis	3	Yes, with modifications	2.5 (1.20)	Yes: 17	9.7
						Yes, with certain reservations: 33	
Clinical Practice Guideline Working Group (ACPG 2008) [40]						No: 50	
Diabetes und Schwangerschaft [Diabetes and pregnancy]/Deutschen Diabetes-Gesellschaft (DDG 2008) [36]	2008/62/GER/German	Diabetes in pregnancy	3	Yes, with modifications	4.9 (1.31)	Yes: 67	11.2
						Yes, with certain reservations: 33	
						No: 0	
Suspected cancer in primary care: Guidelines for investigation, referral and reducing ethnic disparities/New Zealand Guidelines Group (NZGG 2009) [38]	2009/190/NZ/English	Cancer	4	Yes, with modifications	4.3 (1.15)	Yes: 8.3	16.8
						Yes, with certain reservations: 83.3	
						No: 8.3	
Guidelines for the practice of diabetes self-management education/American Association of Diabetes Educators (AADE 2010) [41]	2010/46/USA/English	Diabetes	4	Yes, with modifications	2.8 (1.10)	Yes: 75	20.1
						Yes, with certain reservations: 17	
						No: 8	
Nebennierenrinden-Insuffizienz [Suprarenal gland failure]/Deutschen Gesellschaft für Endokrinologie; Gesellschaft für Kinderheilkunde und Jugendmedizin (DGKJ 2010) [42]	2010/4/GER/German	Suprarenal gland failure	2	No	1.8 (1.11)	Yes: 8	6.8
						Yes, with certain reservations: 17	
						No: 75	
Leitlinie Management der frühen rheumatoiden Arthritis [Guideline managment of early rheumatoid arthritis]/Deutsche Gesellschaft für Rheumatologie (DGR 2011) [37]	2011/163/GER/German	Rheumatoid arthritis	5	Yes	6.7 (0.65)	Yes: 100	13.3
						Yes, with certain reservations: 0	
						No: 0	
Aszites, spontan bakterielle Peritonitis, hepatorenales Syndrom [Ascites, spontaneous bacterial peritonitis, hepatorenal syndrome]/Deutsche Gesellschaft für Verdauungs- undStoffwechselkrankheiten (DGVS 2011) [33]	2011/39/GER/German	Renal failure	5	Yes	6.3 (0.90)	Yes: 92	14.1
						Yes, with certain reservations: 8	
						No: 0	
Leitlinie Management der frühen rheumatoiden Arthritis [Guideline managment of early rheumatoid arthritis]/Deutsche Gesellschaft für Rheumatologie (DGR 2011) [37]	2011/163/GER/German	Rheumatoid arthritis	5	Yes	6.7 (0.65)	Yes: 100	13.3
						Yes, with certain reservations: 0	
						No: 0	
Fieber unklarer Genese [Fever of unknown origin]/Gesellschaft für Kinder- und Jugendrheumatologie Deutschen Gesellschaft für Kinder- und Jugendmedizin (DGKJ 2013) [39]	2013/8/GER/German	Fever	2	No	2.4 (1.00)	Yes: 8	6.8
						Yes, with certain reservations: 17	
						No: 75	
Assessment and Management of Foot Ulcers for People with Diabetes/Registered Nurses’ Association of Ontario (RNAO 2013) [35]	2013/160/USA/English	Diabetic foot lesions	5	Yes	5.5 (1.31)	Yes: 67	18.2
						Yes, with certain reservations: 33	
						No: 0	

*CDN* Canada, *GER* Germany, *NZ* New Zealand, *SD* Standard Deviation, *USA* United States of America

### Primary endpoints on validity and inter-rater reliability of the overall quality rating

The average MiChe quality rating of the guidelines was strongly related to the total AGREE II score (Pearson’s *r* = 0.872; one-tailed *P* < 0.001), as were the recommendations to use the guidelines (Spearman’s ρ = 0.909; one-tailed *P* < 0.001). Both results indicate a high level of validity in the MiChe ratings.

Inter-rater reliability for the overall MiChe quality ratings of the 12 GPs was ICC = 0.755 (one-tailed *P* < 0.001; 95 % CI: 0.572 < ICC < 0.914), indicating good agreement between raters.

### Secondary endpoint for the assessment of the mini-checklist

For the inter-rater reliability of willingness to recommend the guidelines, or “endorsement” for use in practice, Kendall’s W for ordinal ratings was 0.73 (*P* < 0.001), also indicating good agreement between raters.

**Fig. 2:**
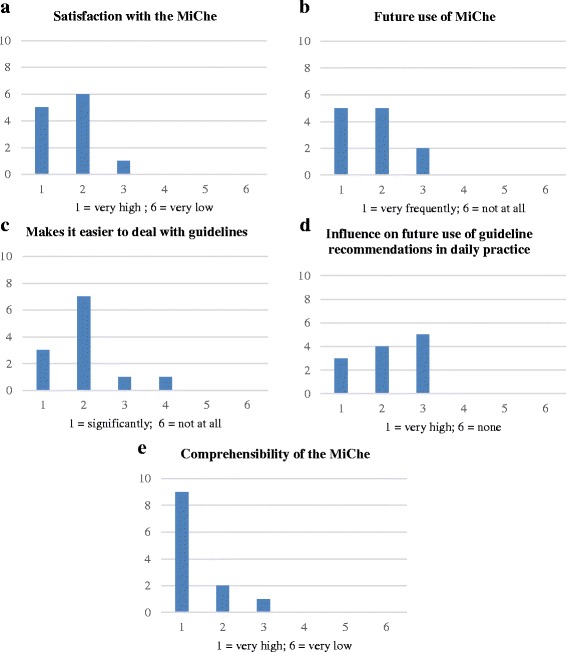
User satisfaction with the Mini-Checklist - 5 queries (**a** Satisfaction with the MiChe; **b** Future use of MiChe; **c** Makes it easier to deal with guidelines; **d** Influence on future use of guideline recommendations in daily practice; **e** Comprehensibility of the MiChe)

Concerning user satisfaction, the mean value for overall satisfaction with the MiChe was 1.7 (SD 0.65) on the six-point Likert scale. As an indicator of future use, the mean value for the MiChe was 2.8 (SD 0.75). The question whether the use of MiChe makes it easier to deal with guidelines resulted in a mean value of 2.0 (SD 0.85). For the question on possible influence on the future implementation of guideline recommendations in daily practice work, the mean value was 2.2 (SD 0.83). For the question on the comprehensibility of the MiChe, the mean value was 1.3 (SD 0.65). For further details see Fig. 2a-e.

The 12 GPs required an average of 12.9 min (SD 9.2) for the MiChe assessment. The mean appraisal time for each guideline ranged from 6.8 to 20.1 min (Table 2).

Eight GPs provided feedback. They would have liked to have a more differentiated assessment scale and mentioned that questions 2 and 3 were rather similar in content. Another suggestion was to add a test question regarding the existence of a structured pocket-version of the guideline for use in practice. Some GPs reckoned that assessments may depend on the language in which the guideline was written and some criticized the questions for their focus on methodological and formal aspects, as they felt this may influence a result even when a recommendation was of proven efficacy. It was further mentioned that the MiChe does not assess the practical usefulness of a guideline on a day-to-day basis.

### Correlation between the domains of AGREE II and the MiChe

The average overall quality rating of the 10 guidelines using MiChe was highly correlated (Pearson’s correlations between 0.74 and 0.87) with the expert ratings in the AGREE II domains II - IV and VI. Correlations for the domains I and V were not statistically significant (Table 3). The pattern of correlations on the level of recommendation with the individual AGREE II domains is very similar. Details of the AGREE II for assessment per domain are shown in Table 4 in the Appendix.

**Table 3:** Correlation between mean AGREE II domain-scores and MiChe overall quality rate/MiChe recommendation for use

AGREE II Domain	Correlation with overall MiChe quality rating	Correlation with MiChe recommendation for use in practice
Domain 1: *Scope and Purpose*	0.55	0.48
Domain 2: *Stakeholder involvement*	0.80*	0.72*
Domain 3: *Rigor of development*	0.87*	0.80*
Domain 4: *Clarity of presentation*	0.74*	0.69*
Domain 5: *Applicability*	0.50	0.44
Domain 6: *Editorial independence*	0.79*	0.74*

* *P* < 0.05

## Discussion

Guidelines have the potential to improve the quality and safety of health care, but are often not used in clinical practice. In order to be helpful, a guideline must be of high methodological quality. The use of comprehensive research-focused instruments such as AGREE II is time-consuming and requires highly qualified personnel. Since practicing physicians are generally very busy, a new rapid-assessment tool (MiChe) was developed to help them evaluate the quality and utility of a guideline quickly and on their own.

This paper presents the results of a validation-study for MiChe [28], as compared to the AGREE II instrument [24]. Ten guidelines that are relevant to general practice and reflect a spectrum of methodological quality ranging from low to high according to an appraisal using the AGREE II instrument were included and assessed using the MiChe by 12 GPs that were inexperienced in guideline appraisal. The study showed a high level of agreement in the quality rating of guidelines between MiChe and AGREE II and recommendations to use the guideline. In addition, inter-rater-reliability for the overall MiChe quality ratings and MiChe recommendation for use in practice were high. With high user satisfaction and a mean time required for guideline assessment of less than 15 min, the MiChe was shown to be suitable for the rapid assessment of guideline quality and utility in practice.

Although the study shows high validity and inter-rater-reliability for the MiChe, it nevertheless has a number of limitations. The validation of the MiChe was performed using the AGREE II instrument as the gold standard for guideline appraisal. AGREE II is the most frequently used instrument for the assessment of methodological guideline quality and has been validated in several studies [43–46]. Nevertheless it remains unclear whether all items and domains of AGREE II contribute equally to the quality of a guideline [25]. The results of our findings that the same individual AGREE II items (II-IV, VI) correlated with both average overall quality ratings and levels of recommendation should not be over-interpreted. The correlations were probably caused by chance, even though it was an interesting result that for domain 3 in particular (rigor of development), the correlation was very high. In addition, we clearly recognize that the questions on the MiChe cannot be seen as independent of the individual AGREE II items. Further empirical studies are needed to find out which items and quality dimensions are essential to the assessment of guideline quality.

Unfortunately we didn’t measure the time it took to assess the guidelines using the AGREE II instrument. However, the AGREE II consortium recommends the use of at least 2 and preferably 4 appraisers and consists of 23 key items organized within 6 domains followed by 2 global rating items. Therefore, we assumed that it requires considerably more time and personnel resources to apply than are typically available to a GP.

Guideline appraisal instruments can be used to assess whether a guideline has been developed in a methodologically accurate and transparent way in accordance with international standards. Guidelines containing adequate information on these topics will therefore be judged to be of high (methodological) quality. But this appraisal is made regardless of whether all recommendations made in the guideline are correct or not. Thus some guidelines of high methodological quality may still contain individual recommendations that are not internally valid in terms of content. Equally, a guideline of low methodological quality may contain recommendations of high content validity [26, 47, 48].

Although the GPs involved in this study were inexperienced in guideline appraisal, they received short, basic training on guideline development and assessment before using the MiChe. A comparison between trained and untrained clinicians with regard to the usability and reliability of the MiChe was not part of this investigation. In addition, convenience sampling of the participants limits generalizability of the results. To achieve wider implementation, future research should assess whether clinicians with no prior training come to the same results as trained clinicians and apply random sampling techniques. To date only the German language version of the MiChe has been validated. It would be useful to know to what extent the use of an English translation of the MiChe would lead to corresponding results.

A large number of manuals and instruments can be used for guideline development and quality assessment. A systematic review carried out by Siering et al in 2013 [27] identified a total of 40 different appraisal tools. Information on quality and validity was only available for 11 of these 40 tools, while detailed information concerning the validation process was reported for only 6. Among these, AGREE II was the most extensively validated instrument [43–46]. In recent years, a number of clinician-focused rapid assessment tools have been developed contemporaneously, and in addition to comprehensive research-focused instruments. Apart from MiChe, these include the iCAHE Guideline Quality Checklist [49], the Global Rating Scale (GRS) of the AGREE Collaboration [50], and the surgeons’ checklist by Coroneos et al. [51]. Of these, MiChe is the only instrument of this type that is available in German and is thus more easily accessible for German speakers. It is also the only tool that has been validated for use in general practice. In 2014, Grimmer et al tested the validity, inter-rater reliability and clinical utility of the iCAHE Guideline Quality Checklist in comparison to the AGREE II instrument [49]. In their study they found a moderate to strong correlation between the iCAHE and the AGREE II scores. A comparison of these four tools was published by Semlitsch et al. in 2015 [30] and showed that, although developed independently, they all focus on a few, broad-based and very similar key questions. They can therefore only give a rudimentary impression of the value of a guideline. They are not intended to provide a comprehensive and detailed guideline appraisal, and include only a broad-based rating system.

## Conclusion

Physicians increasingly use guidelines to gain clinical knowledge. To be dependable, these guidelines need to be prepared using proper methods and to be of sufficiently high quality. The MiChe is a validated rapid-assessment instrument that allows busy physicians to assess the methodical quality of guidelines without the need for experts in guideline appraisal and judge whether a guideline is applicable in patient care or not. It thus increases the likelihood that guideline recommendations will be used in practice and contributes towards sustained improvement in patient health care.

### Declaration and availability statement

The dataset supporting the conclusions of this article are available from the corresponding author on request.

## Appendix

**Table 4:** AGREE II domain-scores and overall quality for assessed guidelines

Guideline	Domain 1	Domain 2	Domain 3	Domain 4	Domain 5	Domain 6	Overall Quality
DEGAM 2006 [34]	0.805	0.722	0.822	0.805	0.687	0.792	6
ACPG 2008 [40]	0.611	0.25	0.104	0.527	0	0	3
DDG 2008 [36]	0.305	0.305	0.416	0.888	0	0.583	3
NZGG 2009 [38]	0.916	0.75	0.562	0.861	0.083	0.625	4
AADE 2010 [41]	0.777	0.472	0.614	0.75	0.562	0.042	4
DGKJ 2010 [42]	0.138	0.027	0.052	0.1666	0	0	2
DGR 2011 [37]	0.694	0.666	0.75	0.777	0.333	0.833	5
DGVS 2011 [33]	0.777	0.638	0.708	0.805	0.145	0.791	5
DGKJ 2013 [39]	0.361	0.222	0.083	0.111	0	0.333	2
RNAO 2013 [35]	0.805	0.527	0.562	0.805	0.541	0.042	5
